# A novel brain functional-structural hybrid analysis to explain the effect of a 6-month psychosocial intervention on resilience in breast cancer

**DOI:** 10.1016/j.ijchp.2025.100639

**Published:** 2025-10-15

**Authors:** Muzi Liang, Jin Zhou, Peng Chen, Wenjing Wu, Yalan Song, Guangyun Hu, Qu Hu, Zhe Sun, Yuanliang Yu, Yuyan Liang, Alex Molassiotis, M. Tish Knobf, Zengjie Ye

**Affiliations:** a Department of Sexual and Reproductive Health, Guangdong Academy of Population Development, Guangzhou, China; b Nursing Department, Southern Medical University Hospital of Integrated Traditional Chinese and Western Medicine, Guangzhou, China; c Basic Medical School, Guizhou University of Traditional Chinese Medicine, Guiyang, China; d School of Life Sciences, State Key Laboratory of Cellular Stress Biology, Xiamen University, Xiamen, China; e Affiliated Cancer Hospital and Institute, Guangzhou Medical University, Guangzhou, China; f School of Nursing, Army Medical University, Chongqing Municipality, China; g Department of Radiation Oncology, Chongqing University Cancer Hospital, Chongqing Municipality, China; h The First Affiliated Hospital, Guangzhou University of Chinese Medicine, Guangzhou, China; i Mental Health Education and Counseling Center, South China University of Technology, Guangzhou, China; j Shenzhen Bao'an Traditional Chinese Medicine Hospital, Guangzhou University of Chinese Medicine, Shenzhen, China; k College of Arts, Humanities and Education, University of Derby, Derby, United Kingdom; l School of Nursing, Yale University, Orange, CT, United States; m School of Nursing, Guangzhou Medical University, Guangzhou, Guangdong Province, China

**Keywords:** Treatment response, Resilience, Intervention, Breast cancer, Brain connectome

## Abstract

**Purposes:**

To explore if pretreatment brain function/structure connectome could explain the response to a psychosocial intervention on resilience in breast cancer.

**Methods:**

Between February 2018 and October 2021, women newly diagnosed with breast cancer were retrospectively enrolled from the *Be Resilient to Breast Cancer* (BRBC) trial and received a supportive-expressive therapy intervention. Baseline Resting-state Functional Magnetic Resonance Imaging (rs-fMRI) combined with Diffusion Tensor Imaging (DTI) were administered and resilience was scored by 10-item Resilience Scale specific to Cancer (RS-SC-10) at baseline and after the intervention (6 months). Response to the supportive intervention on resilience was defined as > 0.5 standard deviation (SD) improvement at 6 months compared to baseline mean resilience score.

**Results:**

A total of 105 patients received intervention. At 6 months, the resilience score improved in 62.9 % (*N* = 66), defined as the Response group. Amygdala (53 %) and Hippocampus (15 %) in rs-fMRI and CorpusCallosum_ForcepsMinor (96 %) in DTI were recognized as the main significant brain regions associated with treatment response.

**Conclusion:**

These preliminary data suggest that neuro-markers of brain function/structure connectome from MR imaging might be useful in evaluating response to behavioral interventions on resilience.

## Introduction

In 2022, more than 2.3 million new cases were recognized in breast cancer (Bray et al., 2024). Psychological distress and coping strategies vary considerably in breast cancer, during and after treatment. Many factors have been identified that adversely affect one’s ability to cope and effect on quality of life (QoL) (Dinapoli et al., 2021). More recently, studies have explored potentially favorable factors such as social support on QoL outcomes or individual characteristics related to coping and patient outcomes, such as resilience (Ding et al., 2024). Resilience, defined as the ability to bounce back after a traumatic event, has been reported as a positive attitudinal approach or characteristic, contributing to a better QoL in women with breast cancer following therapy (Luo et al., 2020; Rutter, 1985). There is some emerging evidence, although sparse, about the construct of resilience and coping of women with breast cancer and brain function/structure connectome. For example, there have been some unique brain regions (i.e., Frontal Medial Cortex, Paracingulate Gyrus) associated with levels of resilience in breast cancer (Liang et al., 2024; Liang et al., 2024b). Biobehavioral interventions to improve QoL in breast cancer span a broad spectrum of psychological support, education, expressive therapy, and stress reduction as examples (Jassim et al., 2023; Schell et al., 2019). Few, if any have included resilience as a primary outcome and there are no known studies that attempt to describe or uncover the etiology, factors or effective components of any given intervention.

With the wide access of non-invasive Functional Magnetic Resonance Imaging (rs-fMRI) and Diffusion Tensor Imaging (DTI), neuromarkers are potential indicators that could be applied to predict treatment response in the resilience interventions (Eaton et al., 2022; Norbury et al., 2023; Tura & Goya-Maldonado, 2023). To explain the association between brain connectome and treatment response in resilience-based interventions, we conducted an analysis using pretreatment MR imaging and changes in resilience between baseline and 6-months in a sample of newly diagnosed women with breast cancer receiving interventions, from the *Be Resilient to Breast Cancer (BRBC)* multicenter historical cohort study (Liang et al., 2024a; Liang et al., 2024). In consideration of quantification of brain functional/structural connectome, data-driven approaches of multi-voxel pattern analysis (MVPA) and correlational tractography (CT) were performed (Nieto-Castanon, 2022; Yeh et al., 2016). We hypothesized that: (1) significant patterns of brain functional/structural connectome would be identified between Response and Non-response groups using MVPA and CT; (2) brain functional/structural connectome could provide additional predicting abilities over the conventional model.

## Method

### Participants

**Fig. 1:**
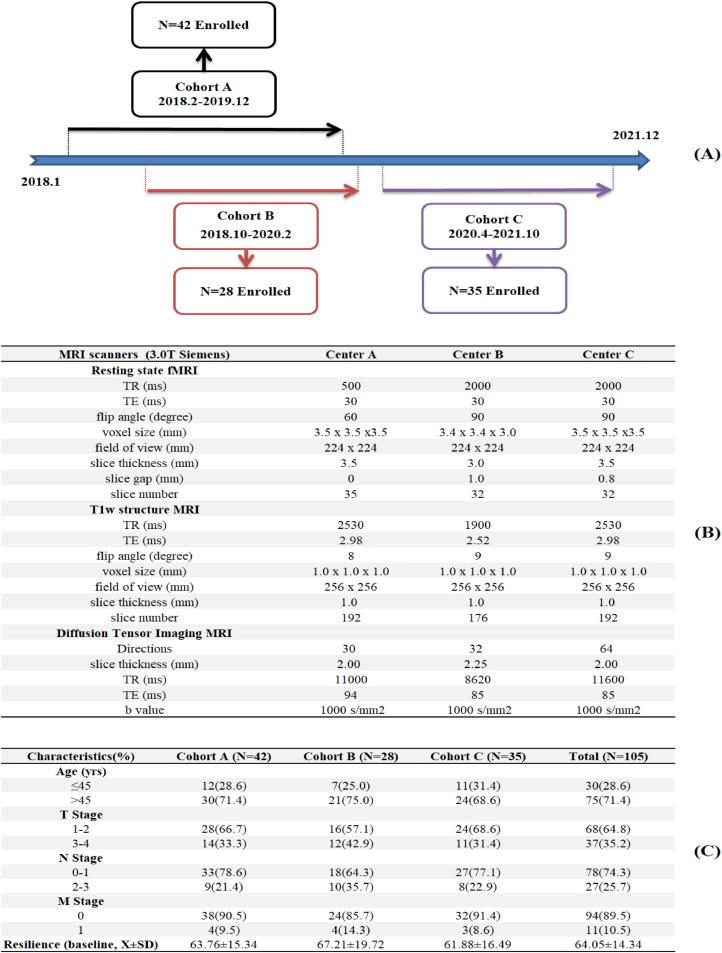
**A:** Timeline for the Enrollment in the SEGT intervention program, **B:** Scanning parameters for rs-fMRI and DTI across three centers, **C:** Demographics for breast cancer patients.

We retrospectively used data from *Be Resilient to Breast Cancer (BRBC)*, a multi-center historical cohort study in southeast China that included participants enrolled from three sites including: Centers A, B and C, which were tertiary hospitals in Guangzhou, Shenzhen and Foshan, respectively. The enrollment was detailed in Fig. 1A. Eligible participants were pathologically confirmed by breast cancer, with sociodemographic and clinical characteristics previously described (Ye et al., 2021, 2020). Informed consent was waived due to anonymous MR images and ethic approval was confirmed in all participating hospitals (202405029).

### Supportive-expressive group therapy (SEGT)

There were 11 sessions, with each having two components (education and group discussion), delivered once per week in the first two months as well as once per month in the following four months (Ye et al., 2021, 2020). Education consists of multidiscipline topics, for instance, surgery, lymphedema, mindfulness and Chinese medicine. For group discussion, trained breast cancer survivors were invited to participate as mentors to provide peer support. Of the 11 sessions, participants who attended a minimum of 2 sessions were included in the analysis. Details about SEGT were described in Table S1.

### Treatment response of resilience

It is measured by 10-item Resilience Scale Specific to Cancer (RS-SC-10) (Liang et al., 2022; Ye et al., 2018). The total score of RS-SC-10 ranges from 10 to 50 and is standardized for the estimation of changes. The Cronbach’s coefficient was 0.86 in the present study.

0.5 was defined as a moderate effect size according to Cohen’s d and a 0.5 standard deviation (SD) increasement was chosen as the primary outcome in the current study (Diener, 2010). Compared to those at baseline, participants with > 0.5 SD improvement in resilience total scores were defined as the Response group while others were classified into Non-response group. Similar procedures were calculated for the distribution method of minimal clinically important difference (MCID) in different clinical trials (Javeed et al., 2023; Kunze et al., 2021; Norman et al., 2003).

### MR image acquisition

Imaging data were collected using 3.0 T Siemens scanners at three centers. The details about the scanning parameters in each center were described in Fig. 1B Automated file name and manual image quality control rating were performed to ensure high-quality data across different centers. Spatial preprocessing procedures for rs-fMRI and DTI has been detailed elsewhere (Liang et al., 2024; Liang et al., 2024b). There is significant heterogeneity in imaging parameters across sites, which could impact the validity of pooled analyses. We addressed parameter heterogeneity as follows: First, rs-fMRI harmonization. We used band-pass filtering (0.01–0.1 Hz) to standardize frequency ranges across sites and Framewise Displacement (FD) regression was performed to remove motion artifacts correlated with TR differences (Power et al., 2014). In addition, all functional images were resliced to 3.5 mm isotropic voxels and slice gap effects were minimized via spatial smoothing (6 mm FWHM) and slice-timing correction, eliminating inter-slice temporal offsets. These procedures standardize temporal dynamics of rs-fMRI across different sites. Second, DTI protocol variability. We used Generalized Q-Sampling Imaging (GQI) in DSI Studio for direction-independent reconstruction, which computed spin distribution functions (SDF) robust to sampling density (Yeh et al., 2010). In addition, we derived normalized quantitative anisotropy (NQA) for tractography, which was shown to stabilize with ≥30 directions (Takao et al., 2011). Further, we re-ran analyses using site-specific direction subsets (30 directions for all sites) and results correlated at *r* = 0.95 with full data, indicating robust findings. These comprehensive procedures ensured that observed effects reflect true biological signals rather than technical variability.

### Data analysis

First, principal components analysis (PCA) was utilized to dimensionality reduction in the rs-fMRI dataset and a conservative ratio of 40:1 (*N* = 3) was set to determine the optimal component in the total cohort (*N* = 105). Then, according to the treatment response of resilience (Response Vs. Non-response), a multi-voxel pattern analysis (MVPA) was performed using Automated Anatomical Labelling (AAL) to identify significant functional brain regions after controlling the confounders (i.e., TNM staging, research site, etc.) using Generalized Linear Model (GLM) (Whitfield-Gabrieli & Nieto-Castanon, 2012). Second, using quantitative anisotropy (QA) and deterministic fiber tracking algorithm, correlational tractography (CT) was performed to recognize significant structural brain fibers against the response of the intervention on resilience (Response vs. Non-response) after controlling for potential confounders (i.e., TNM staging, research site, etc.) using GLM (Yeh, 2020). To ensure robust tracking fibers, the threshold was set at 2.0T and the minimum length was set at 20 voxels, followed by 4000 permutations. Third, Net Reclassification Improvement (NRI) combined with Integrated Discrimination Improvement (IDI) were calculated when Blood-oxygen-level dependent (BOLD) in rs-fMRI and QA in DTI were incorporated into the prediction model. According to the TRIPOD guideline, decision curves and clinical impact curves were also estimated (Collins et al., 2015). CONN software, SPM 12 and DSI Studio were used for brain connectome analysis and R software was used for prediction models.

## Results

### Sample

In Fig. 1C, Cohort A (*N* = 42), Cohort B (*N* = 28) and Cohort C (*N* = 35) completed the pretreatment MR imaging as well as resilience questionnaires at baseline and 6 months. The majority of participants (64.8 %) were diagnosed with early stage of breast cancer (I-II). On a standardized scale of 0–100, the baseline resilience score was 64.05 (SD = 14.34) and 62.9 % (*N* = 66) was defined as the Response group.

### Significant functional connectome in rs-fMRI

**Fig. 2:**
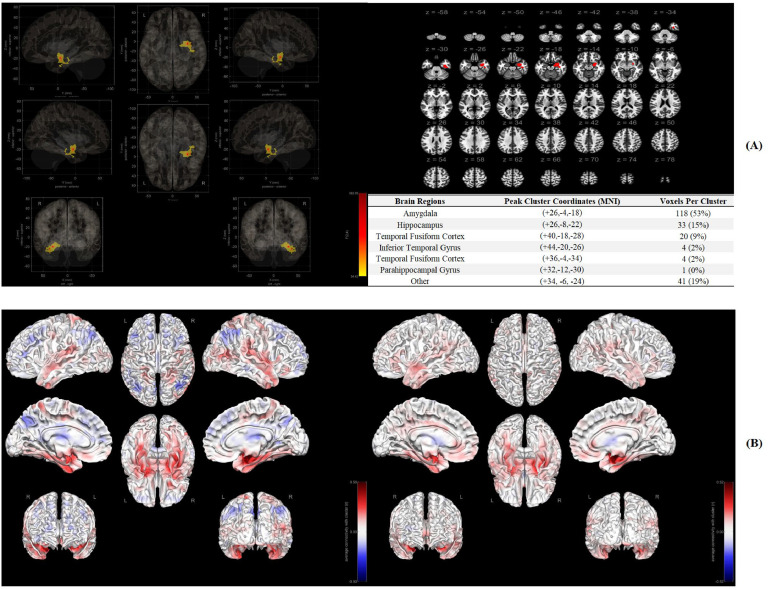
**A:** Significant brain regions in the functional connectome, **B:** Seed-to-voxel associations in the Response/Non-response groups.

In Fig. 2A, Amygdala (118 voxels, 53 %), Hippocampus (33 voxels, 15 %) and Temporal Fusiform Cortex (20 voxels, 9 %) were recognized as significant brain regions (ROI) associated with behavioral intervention response on resilience. In Fig. 2B, a stronger association between ROI and other brain regions was identified in Response group of the intervention compared to the participants who did not have *a* > 0.5SD improvement in their mean resilience scores (Non-response group).

### Significant structural connectome in DTI

**Fig. 3:**
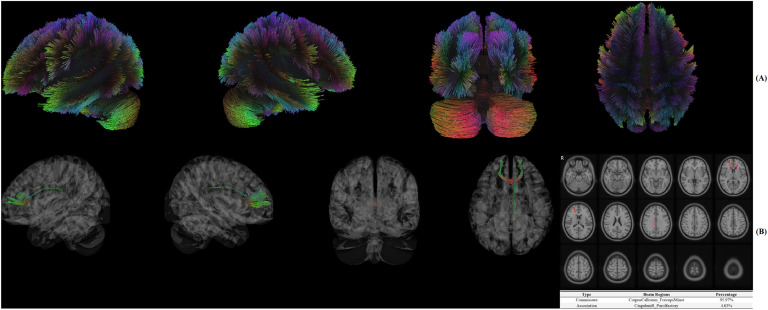
**A:** Reconstruction for the white fibers using deterministic fiber tracking algorithm, **B:** Significant white fibers in the structural connectome.

In Fig. 3A, white fibers were reconstructed using deterministic fiber tracking algorithm. In Fig. 3B, CorpusCallosum_ForcepsMinor (95.97 %) and CingulumR_Parolfactory (4.03 %) were identified as significant brain fibers associated with treatment response of resilience.

### Prediction models for treatment response of resilience

**Fig. 4:**
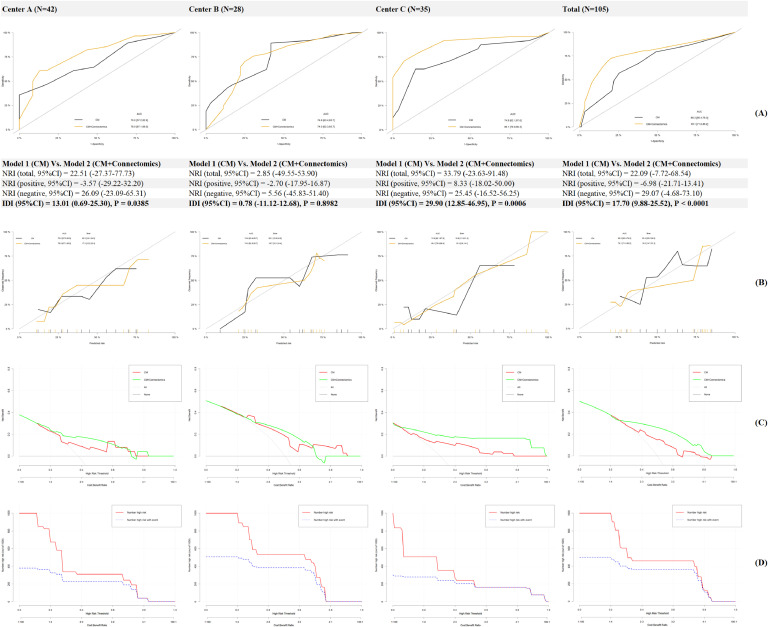
**A:** AUC, NRI and IDI for Model 1 (CM) Vs. Model 2 (CM+Connectomics), **B:** Calibration curves for different prediction models, **C:** Decision curve analysis for different prediction models, **D:** Clinical Impact Curves for Model 2 (CM+Connectomics).

In Fig. 4A, AUC in Model 1(Conventional Model) increased from 68.3–74.9 % to 74.0–89.1 % in Model 2 (Conventional Model+ Connectomics) when brain functional/structural connectome was incorporated. In addition, the NRI and IDI in the total cohort were 22.09 % and 17.70 % respectively, indicating a significant improvement in the prediction ability. In Fig. 4B, a less Brier score was calculated in Model 2 compared to that in Model 1 (18.0 Vs. 22.4), indicating a better fitting when the brain connectome was added as additional predictors. In Fig. 4C, compared to that in Model 1, a higher net benefit was recognized in patients with moderate to high risk using Model 2, and the clinical impact curve was described for Model 2 in Fig. 4D

## Discussion

It is the first study to explore if pretreatment brain functional/structural connectome was associated with or could explain treatment response to a behavioral supportive care intervention delivered in the first 6 months after diagnosis of breast cancer on resilience. While there are a few studies that looked at outcomes of a psychotherapy intervention for MDD, there are no other studies that incorporated resilience as a study variable in the context of brain imaging and function (Tura & Goya-Maldonado, 2023). In the present study, Amygdala, Hippocampus and Temporal Fusiform Cortex in rs-fMRI as well as CorpusCallosum_ForcepsMinor and CingulumR_Parolfactory in DTI were recognized as significant brain regions (ROI) associated with treatment response. When brain functional/structural connectome was included into the prediction model, a significant improvement in the prediction ability was recognized.

First, significant patterns of brain functional/structural connectome were identified between response and non-response groups using data-driven methods of MVPA and CT. Thus, hypothesis 1 was validated here. As for functional connectome, resilience was reported to be associated with Medial Prefrontal Cortex (mPFC) function in downregulation of limbic regions (i.e., amygdala) in general populations, which could be partially replicated in the present study (Maier & Watkins, 2010). Further, Ventrolateral Prefrontal Cortex (vlPFC) combined with Ventromedial Prefrontal Cortex (vmPFC) were often reported to be associated with the treatment response of a psychotherapy in MDD (Crowther et al., 2015; Dunlop et al., 2017). However, these findings could not be replicated in the current study with a different outcome of resilience. The potential reasons were complex in consideration of the chosen methods, sample size, race, populations, etc. As for structural connectome, previous study investigating the association between psychotherapy and structural connectome was limited while frontal and parietal regions (i.e., thalamus, angular gyrus) were believed to be the main brain areas in MDD, which was partially replicated in the present study (Wang et al., 2013). However, out of our expectations, the significant brain regions recognized by MVPA in the functional connectome were not consistent with those identified by CT in structural connectome, although similar phenomenon was also identified in previous research (Kesler et al., 2017). The main reason might be attributed to methodological divergence as functional (multivariate pattern analysis) and structural (tractography) methods capture distinct properties (network-wide vs. localized effects). Without a priori ROI-ROI theoretical hypothesis in the data-driven methods for rs-fMRI and DTI in the current study, a maximized sensitivity to significant brain regions was achieved by MVPA at the cost of decreasing specificity. For example, 41 voxels (19 %) in MVPA could not be anatomically labelled in MNI. Similarly, white fibers were reconstructed by deterministic fiber tracking algorithm in CT and the true axonal connections may not be “real” (Maier-Hein et al., 2017). As for DTI constraints (e.g., crossing fibers, false positives), deterministic vs. probabilistic tractography comparisons are recommended. Thus, in consideration of small sample size in the current study, Type I errors should be considered although FDR for significant findings was controlled. In addition, the corpus callosum (structural) and limbic connectivity (functional) may reflect independent pathways to resilience which should be confirmed and replicated in future research.

Second, as for a validation method for hypothesis 1, the new prediction model incorporating brain functional/structural connectome provided a significant 17.70 % of IDI over the conventional model with approximately 74.0–89.1 % accuracy. Thus, hypothesis 2 was also confirmed here. In consideration of wide access and non-invasiveness of MR imaging worldwide, these neuromarkers could be utilized for precision intervention and management in breast cancer. However, fMRI is expensive and research about cost-effectiveness of MR imaging in resilience-based interventions should be further performed (Burton et al., 2014). In addition, due to the compromised statistical power derived from a small sample, many confounders including education, income, family function, etc., were not controlled in the regressions to avoid local optima which would affect the predicting accuracy (Duffy et al., 2022). At last, 0.5 SD improvement of resilience as the cut-off of treatment response was also arbitrary without established reference and more cut-offs should be tried in future MR imaging research.

## Limitations

First, only pretreatment MR imaging was collected and the lack of fMRI data at 6 months limits any interpretation of a longitudinal association between changes in resilience and changes in brain functional/structural connectome. Second, the sample size is relatively small (*N* = 105) and unevenly distributed across the three centers (Cohort *A* = 42; *B* = 28; *C* = 35). This may limit statistical power, particularly for subgroup analyses, such as those based on TNM staging. The prediction abilities of brain functional/structural connectome were not stable across three centers and the potential reason is attributed to low statistical power or difference among patient cohort characteristics. Greater variability in resilience among patients, particularly in Cohort B, suggests the need for further exploration of contextual factors (e.g., socioeconomic status, support systems) that may moderate intervention effects. Third, measurement errors were not considered in the calculation of resilience as an intervention response. PCA, item response theory (IRT) and cognitive diagnosis models (CDMs) could be explored to achieve a more precise anchor-based calculation (Ma et al., 2023; Terluin et al., 2023). At last, the ability to explain or predict treatment response by functional and structural connectome were not estimated separately which should be further explored with a larger sample size in the multimodal research.

## Conclusion

There is a connection between brain functional/structural connectome and response to a supportive care intervention on resilience in breast cancer. The neuromarkers of the fMRI offer a potential to confirm the effect of biobehavioral interventions on resilience and also other patient reported outcomes.

## Registration number

It is registered in ClinicalTrials.gov (NCT03026374).

## Data availability statement

The data that support the findings of this study are available on request from the corresponding author. The data are not publicly available due to privacy or ethical restrictions.

## Declaration of competing interest

All other authors report no biomedical financial interests or potential conflicts of interest.

## Appendix Supplementary materials

Supplementary Description: Table S1. The details about the design of Supportive-expressive group therapy (SEGT). Image, application 1

## References

[bib0001] Bray F., Laversanne M., Sung H., Ferlay J., Siegel R.L., Soerjomataram I., Jemal A. (2024). Global cancer statistics 2022: GLOBOCAN estimates of incidence and mortality worldwide for 36 cancers in 185 countries. CA: A Cancer Journal for Clinicians.

[bib0036] Burton K.R., Perlis N., Aviv R.I., Moody A.R., Kapral M.K., Krahn M.D., Laupacis A. (2014). Systematic review, critical appraisal, and analysis of the quality of economic evaluations in stroke imaging. Stroke; A Journal of Cerebral Circulation.

[bib0028] Collins G.S., Reitsma J.B., Altman D.G., Moons K.G. (2015). Transparent reporting of a multivariable prediction model for individual prognosis or diagnosis (TRIPOD): The TRIPOD statement. BMJ (Clinical Research Ed.).

[bib0031] Crowther A., Smoski M.J., Minkel J., Moore T., Gibbs D., Petty C., Bizzell J., Schiller C.E., Sideris J., Carl H., Dichter G.S. (2015). Resting-state connectivity predictors of response to psychotherapy in major depressive disorder. Neuropsychopharmacol.

[bib0019] Diener M.J. (2010).

[bib0002] Dinapoli L., Colloca G., Di Capua B., Valentini V. (2021). Psychological aspects to consider in breast cancer diagnosis and treatment. Current Oncology Reports.

[bib0003] Ding X., Zhao F., Wang Q., Zhu M., Kan H., Fu E., Wei S., Li Z. (2024). Effects of interventions for enhancing resilience in cancer patients: A systematic review and network meta-analysis. Clinical Psychology Review.

[bib0037] Duffy G., Clarke S.L., Christensen M., He B., Yuan N., Cheng S., Ouyang D. (2022). Confounders mediate AI prediction of demographics in medical imaging. NPJ Digital Medicine.

[bib0032] Dunlop B.W., Rajendra J.K., Craighead W.E., Kelley M.E., McGrath C.L., Choi K.S., Kinkead B., Nemeroff C.B., Mayberg H.S. (2017). Functional connectivity of the subcallosal cingulate cortex and differential outcomes to treatment with cognitive-behavioral therapy or antidepressant medication for major depressive disorder. The American Journal of Psychiatry.

[bib0010] Eaton S., Cornwell H., Hamilton-Giachritsis C., Fairchild G. (2022). Resilience and young people's brain structure, function and connectivity: A systematic review. Neuroscience and Biobehavioral Reviews.

[bib0008] Jassim G.A., Doherty S., Whitford D.L. (2023). Khashan AS. Psychological interventions for women with non-metastatic breast cancer. The Cochrane Database of Systematic Reviews.

[bib0021] Javeed S., Greenberg J.K., Plog B., Zhang J.K., Yahanda A.T., Dibble C.F., Khalifeh J.M., Ruiz-Cardozo M., Lavadi R.S., Molina C.A., Santiago P., Agarwal N., Pennicooke B.H., Ray W.Z. (2023). Clinically meaningful improvement in disabilities of arm, shoulder, and hand (DASH) following cervical spine surgery. The Spine Journal : Official Journal of the North American Spine Society.

[bib0034] Kesler S.R., Adams M., Packer M., Rao V., Henneghan A.M., Blayney D.W., Palesh O. (2017). Disrupted brain network functional dynamics and hyper-correlation of structural and functional connectome topology in patients with breast cancer prior to treatment. Brain and Behavior.

[bib0020] Kunze K.N., Bart J.A., Ahmad M., Nho S.J., Chahla J. (2021). Large heterogeneity among minimal clinically important differences for hip arthroscopy outcomes: a systematic review of reporting trends and quantification methods. Arthroscopy.

[bib0018] Liang M.Z., Chen P., Molassiotis A., Jeon S., Tang Y., Hu G.Y., Zhu Y.F., Sun Z., Yu Y.L., Knobf M.T., Ye Z.J. (2022). Measurement invariance of 10-item resilience scale specific to cancer in Americans and Chinese: A propensity score-based multidimensional item response theory analysis. Asia Pacific Journal of Oncology Nursing.

[bib41] Liang M.Z., Chen P., Tang Y., Liang Y.Y., Li S.H., Hu G.Y., Sun Z., Yu Y.L., Molassiotis A., Knobf M.T., Ye Z.J. (2024). Associations between brain structural connectivity and 1-year demoralization in breast cancer: A longitudinal diffusion tensor imaging study. Depress Anxiety.

[bib40] Liang M.Z., Zhou J., Chen P, Song Y, Li S.H., Liang Y.Y., Hu G.Y., Hu Q., Sun Z., Yu Y.L., Molassiotis A., Knobf M.T., Ye Z.J. (2024). A longitudinal correlational study of psychological resilience, depression disorder, and brain functional-structural hybrid connectome in breast cancer. Depress Anxiety.

[bib0006] Liang M.Z., Tang Y., Chen P., Tang X.N., Knobf M.T., Hu G.Y., Sun Z., Liu M.L., Yu Y.L., Ye Z.J. (2024). Brain connectomics improve prediction of 1-year decreased quality of life in breast cancer: A multi-voxel pattern analysis. European Journal of Oncology Nursing : The Official Journal of European Oncology Nursing Society.

[bib0007] Liang M.Z., Chen P., Tang Y., Tang X.N., Molassiotis A., Knobf M.T., Liu M.L., Hu G.Y., Sun Z., Yu Y.L., Ye Z.J. (2024). Brain connectomics improve the prediction of high-risk depression profiles in the first year following breast cancer diagnosis. Depression and Anxiety.

[bib0005] Luo D., Eicher M., White K. (2020). Individual resilience in adult cancer care: A concept analysis. International Journal of Nursing Studies.

[bib0039] Ma C., Ouyang J., Xu G. (2023). Learning latent and hierarchical structures in cognitive diagnosis models. Psychometrika.

[bib0030] Maier S.F., Watkins L.R. (2010). Role of the medial prefrontal cortex in coping and resilience. Brain Research.

[bib0035] Maier-Hein K.H., Neher P.F., Houde J.C., Côté M.A., Garyfallidis E., Zhong J., Chamberland M., Yeh F.C., Lin Y.C., Ji Q., Reddick W.E., Glass J.O., Chen D.Q., Feng Y., Gao C., Wu Y., Ma J., He R., Li Q., Westin C.F., Deslauriers-Gauthier S., González J.O.O., Paquette M., St-Jean S., Girard G., Rheault F., Sidhu J., Tax C.M.W., Guo F., Mesri H.Y., Dávid S., Froeling M., Heemskerk A.M., Leemans A., Boré A., Pinsard B., Bedetti C., Desrosiers M., Brambati S., Doyon J., Sarica A., Vasta R., Cerasa A., Quattrone A., Yeatman J., Khan A.R., Hodges W., Alexander S., Romascano D., Barakovic M., Auría A., Esteban O., Lemkaddem A., Thiran J.P., Cetingul H.E., Odry B.L., Mailhe B., Nadar M.S., Pizzagalli F., Prasad G., Villalon-Reina J.E., Galvis J., Thompson P.M., Requejo F.S., Laguna P.L., Lacerda L.M., Barrett R., Dell'Acqua F., Catani M., Petit L., Caruyer E., Daducci A., Dyrby T.B., Holland-Letz T., Hilgetag C.C., Stieltjes B., Descoteaux M. (2017). The challenge of mapping the human connectome based on diffusion tractography. Nature Communications.

[bib0013] Nieto-Castanon A., Filippi M. (2022). fMRI techniques and protocols.

[bib0011] Norbury A., Seeley S.H., Perez-Rodriguez M.M., Feder A. (2023). Functional neuroimaging of resilience to trauma: Convergent evidence and challenges for future research. Psychological Medicine.

[bib0022] Norman G.R., Sloan J.A., Wyrwich K.W. (2003). Interpretation of changes in health-related quality of life: The remarkable universality of half a standard deviation. Medical Care.

[bib0023] Power J.D., Mitra A., Laumann T.O., Snyder A.Z., Schlaggar B.L., Petersen S.E. (2014). Methods to detect, characterize, and remove motion artifact in resting state fMRI. Neuroimage.

[bib0004] Rutter M. (1985). Resilience in the face of adversity. Protective factors and resistance to psychiatric disorder. The British Journal of Psychiatry.

[bib0009] Schell L.K., Monsef I., Wöckel A., Skoetz N. (2019). Mindfulness-based stress reduction for women diagnosed with breast cancer. The Cochrane Database of Systematic Reviews.

[bib0025] Takao H., Hayashi N., Ohtomo K. (2011). Effect of scanner in asymmetry studies using diffusion tensor imaging. Neuroimage.

[bib0038] Terluin B., Koopman J.E., Hoogendam L., Griffiths P., Terwee C.B., Bjorner J.B. (2023). Estimating meaningful thresholds for multi-item questionnaires using item response theory. Quality of Life Research : An International Journal of Quality of Life Aspects of Treatment, Care and Rehabilitation.

[bib0012] Tura A., Goya-Maldonado R. (2023). Brain connectivity in major depressive disorder: A precision component of treatment modalities?. Translational Psychiatry.

[bib0029] Tura A., Goya-Maldonado R. (2023). Brain connectivity in major depressive disorder: A precision component of treatment modalities?. Translational Psychiatry.

[bib0033] Wang T., Huang X., Huang P., Li D., Lv F., Zhang Y., Zhou L., Yang D., Xie P. (2013). Early-stage psychotherapy produces elevated frontal white matter integrity in adult major depressive disorder. PLoS ONE.

[bib0026] Whitfield-Gabrieli S., Nieto-Castanon A. (2012). Conn: A functional connectivity toolbox for correlated and anticorrelated brain networks. Brain Connectivity.

[bib0017] Ye Z.J., Liang M.Z., Li P.F., Sun Z., Chen P., Hu G.Y., Yu Y.L., Wang S.N., Qiu H.Z. (2018). New resilience instrument for patients with cancer. Quality of Life Research : An International Journal of Quality of Life Aspects of Treatment, Care and Rehabilitation.

[bib0016] Ye Z.J., Zhang Z., Zhang X.Y., Tang Y., Liang J., Sun Z., Liang M.Z., Yu Y.L. (2020). Effectiveness of adjuvant supportive-expressive group therapy for breast cancer. Breast Cancer Research and Treatment.

[bib0015] Ye Z.J., Zhang Z., Tang Y., Liang J., Sun Z., Hu G.Y., Liang M.Z., Yu Y.L. (2021). Resilience patterns and transitions in the Be Resilient To Breast Cancer trial: An exploratory latent profile transition analysis. Psycho-oncology.

[bib0027] Yeh F.C. (2020). Shape analysis of the human association pathways. Neuroimage.

[bib0014] Yeh F.C., Badre D., Verstynen T. (2016). Connectometry: A statistical approach harnessing the analytical potential of the local connectome. Neuroimage.

[bib0024] Yeh F.C., Wedeen V.J., Tseng W.Y. (2010). Generalized q-sampling imaging. IEEE Transactions on Medical Imaging.

