# Supplementary material for: A novel brain functional-structural hybrid analysis to explain the effect of a 6-month psychosocial intervention on resilience in breast cancer
Source: Int J Clin Health Psychol. 2025 Oct 15;25(4):100639. doi: 10.1016/j.ijchp.2025.100639 (PMC12550284; doi:10.1016/j.ijchp.2025.100639)
Supplement: Supplementary file 1 — Supplementary Description: Table S1. The details about the design of Supportive-expressive group therapy (SEGT). [file mmc1.docx]

**Table S1. The details about the design of Supportive-expressive group therapy (SEGT)**

| **Week** | **Theme** | **Activities** | **Participants** |
| --- | --- | --- | --- |
| **1** | **Introduction** | 1.Introduction of the program by project nurses  2.Mentors and mentees get to know each other  3. Group discussion among mentors and mentees | Nurses, mentors and mentees |
| **2** | **Surgery and treatment** | 1.Presentation of breast cancer post-treatment issues by oncologists  2.Sharedexperiencesofbreast reconstruction by mentors  3.Group discussion among mentors and mentees | Oncologists, nurses, mentors and mentees |
| **3** | **Physical therapy** | 1.Presentation of lymphokinetic motion and pressure gradient by physical therapists  2.Tips from mentors to get through lymphedema  3.Group discussion among mentors and mentees | Physical therapists, nurses, mentors and mentees |
| **4** | **Emotional distress** | 1.Presentation of anxiety, depression, illness uncertainty, etc. by psychologists  2.Shared trajectory of emotional distress as perceived by mentors and tips to get over them  3.Group discussion among mentors and mentees | Psychologists, nurses, mentors and mentees |
| **5** | **Nausea, diet and nutrition** | 1.Presentation of diet and nutrition after surgery by dietitians  2. Recipes recommended by mentors to deal with nausea induced by chemotherapy  3.Group discussion among mentors and mentees | Dietitians, nurses, mentors and mentees |
| **6** | **Traditional Chinese Medicine and Taichi practice** | 1.Presentation of Traditional Chinese Medicine (TCM) and practice of Taichi by Chinese medical practitioners  2.Other tips recommended by mentors  3.Group discussion among mentors and mentees | Chinese medical practitioners, nurses, mentors and mentees |
| **7** | **Music and Relaxation techniques** | 1.Presentation of emotion management and positive mind therapy by psychologists  2.Learning Chinese five-element music as taught by musical therapists; the mentees were instructed to engage in home practice  3.Other relax tips as recommended by mentors  4.Group discussion among mentors and mentees | Psychologists, music therapists, nurses, mentors and mentees |
| **8** | **Sexuality issues and review the program** | 1.Presentation of how to regain sexuality by physicians and psychologists  2.Help mentees review the program by nurses and mentors  3. Group discussion among mentors and mentees | Physicians, psychologists, nurses, mentors and mentees |
| **12** | **Restoration** | 1.Presentation by mentees of what they understand and what problems have occurred in the last 3 months  2.Help mentees review the program and solve specific problems by related medical staffs  3.Group discussion among mentors and mentees  4.Set a specific date for the next class | Oncologists, physical therapists, dietitians, Chinese medical practitioners, psychologists, nurses, mentors and mentees |
| **16** | **To be better** | 1.Presentation by mentees of what they understand and what problems have occurred in the last 6 months  2.Help mentees review the program by nurses and mentors and solve specific problems by related medical staff  3.Group discussion among mentors and mentees  4.Set a specific date for the next class | Oncologists, physical therapists, dietitians, Chinese medical practitioners, psychologists, nurses, mentors and mentees |
| **24** | **Renewal** | 1.Sharing by graduates of the program and family members  2.Ask mentees if they want to be mentors for other new breast cancer patients | Oncologists, physical therapists, dietitians, Chinese medical practitioners, psychologists, nurses, mentors and mentees |
